# Unraveling the chromosome 17 patterns of FISH in interphase nuclei: an in-depth analysis of the *HER2* amplicon and chromosome 17 centromere by karyotyping, FISH and M-FISH in breast cancer cells

**DOI:** 10.1186/1471-2407-14-922

**Published:** 2014-12-07

**Authors:** Milena Rondón-Lagos, Ludovica Verdun Di Cantogno, Nelson Rangel, Teresa Mele, Sandra R Ramírez-Clavijo, Giorgio Scagliotti, Caterina Marchiò, Anna Sapino

**Affiliations:** Department of Medical Sciences, University of Turin, Via Santena 7, 10126 Turin, Italy; Department of Laboratory Medicine, Azienda Ospedaliera Città della Salute e della Scienza di Torino, Corso Bramante 88, 1026 Tutin, Italy; Departments of Oncology and Clinical and Biological Sciences, University of Turin, San Luigi Hospital, Orbassano, Turin, Italy; Natural and Mathematical Sciences Faculty, Universidad del Rosario, Bogotá, Colombia

**Keywords:** Breast cancer, Chromosome 17, Polysomy, CEP17, *HER2*, *TOP2A*, *STARD3*, M-FISH, Chromosomal rearrangements

## Abstract

**Background:**

In diagnostic pathology, *HER2* status is determined in interphase nuclei by fluorescence *in situ* hybridization (FISH) with probes for the *HER2* gene and for the chromosome 17 centromere (CEP17). The latter probe is used as a surrogate for chromosome 17 copies, however chromosome 17 (Chr17) is frequently rearranged. The frequency and type of specific structural Chr17 alterations in breast cancer have been studied by using comparative genomic hybridization and spectral karyotyping, but not fully detailed. Actually, balanced chromosome rearrangements (e.g. translocations or inversions) and low frequency mosaicisms are assessable on metaphases using G-banding karyotype and multicolor FISH (M-FISH) only.

**Methods:**

We sought to elucidate the CEP17 and *HER2* FISH patterns of interphase nuclei by evaluating Chr17 rearrangements in metaphases of 9 breast cancer cell lines and a primary culture from a triple negative breast carcinoma by using G-banding, FISH and M-FISH.

**Results:**

Thirty-nine rearranged chromosomes containing a portion of Chr17 were observed. Chromosomes 8 and 11 were the most frequent partners of Chr17 translocations. The lowest frequency of Chr17 abnormalities was observed in the HER2-negative cell lines, while the highest was observed in the HER2-positive SKBR3 cells. The MDA-MB231 triple negative cell line was the sole to show only non-altered copies of Chr17, while the SKBR3, MDA-MB361 and JIMT-1 HER2-positive cells carried no normal Chr17 copies. True polysomy was observed in MDA-MB231 as the only Chr17 alteration. In BT474 cells polysomy was associated to Chr17 structural alterations. By comparing M-FISH and FISH data, in 8 out of 39 rearranged chromosomes only CEP17 signals were detectable, whereas in 14 rearranged chromosomes *HER2* and *STARD3* genes were present without CEP17 signals. *HER2* and *STARD3* always co-localized on the same chromosomes and were always co-amplified, whereas *TOP2A* also mapped to different derivatives and was co-amplified with *HER2* and *STARD3* on SKBR3 cells only.

**Conclusion:**

The high frequency of complex Chr17 abnormalities suggests that the interpretation of FISH results on interphase nuclei using a dual probe assay to assess gene amplification should be performed “with caution”, given that CEP17 signals are not always indicative of normal unaltered or rearranged copies of Chr17.

## Background

Chromosome17 (Chr17) is the second most gene-dense chromosome in the human genome [1], containing many genes central to breast cancer development and progression, including oncogenes (*HER2, TOP2A, STARD3, TAU*)*,* tumor suppressor genes *(TP53, BRCA1, HIC-1)* and DNA double-strand break repair genes (*RDM1*) [2–7]. In particular, the *HER2* gene mapping to 17q11-q12 is amplified in 15-20% of all breast cancers [8], it is a prognostic marker for aggressiveness [8] and predicts the response to anti-HER2 agents [8]. An accurate and definitive reporting of *HER2* status is thus essential for appropriate treatment determination. Fluorescence *in situ* hybridization (FISH) with dual probes for *HER2* and for the Chr17 centromere (CEP17) is the technique most frequently used in diagnostic pathology to determine the *HER2* gene status in interphase nuclei. The correction of *HER2* gene copy number using CEP17 signals is required to account for Chr17 polysomy. However, by microarray-based comparative genomic hybridization (CGH) analysis we have recently provided the first direct evidence that true Chr17 polysomy is a rare event in breast cancer [9]. Indeed, a number of CEP17 copies greater than 3 detected by FISH analysis is frequently related to either a gain or amplification of the centromere region, providing another line of evidence that Chr17 usually displays very complex rearrangements.

CGH, loss of heterozygosity (LOH), and molecular genetics studies have shown that Chr17 is rearranged in at least 30% of breast tumors [1, 10, 11] and presents a number of rearrangement breakpoints mapping to either its short or long arm. In particular, 17p is principally involved in losses, whereas CGH on 17q shows complex combinations of overlapping gains and losses [1, 12]. In addition, CGH and spectral karyotyping (SKY) studies have shown that Chr17 is one of the chromosomes most frequently involved in translocations [13]. However the frequency and type of specific structural Chr17 alterations in breast cancer have not been fully detailed. For example, balanced chromosome rearrangements (e.g. translocations or inversions) and low frequency mosaicisms are assessable on metaphases using G-banding karyotype and multicolor fluorescence *in situ* hybridization (M-FISH) only.

The complexity of Chr17 rearrangements calls into question the accuracy of *HER2*/CEP17 ratios evaluated on interphase nuclei for diagnostic purposes. Indeed, unsuspected Chr17 rearrangements may be contributing to the equivocal results following *in situ* hybridization testing, which account for about 10% of all IHC score 2+ carcinomas [14].

The aim of this study was to assess numerical alterations and structural rearrangements of Chr17 in breast cancer cells and to elucidate how these alterations may impact on the *HER2*/CEP17 FISH results on interphase nuclei.

## Methods

### Cell lines

Nine established breast cancer cell lines [MCF7, T47D, ZR-75-1 (estrogen receptor positive (ER+), *HER2* not amplified), BT474, MDA-MB361 (ER+, *HER2* amplified), SKBR3, JIMT-1 and KPL4 (ER-, *HER2* amplified) and MDA-MB231 (ER-, *HER2* not amplified)] were obtained from the American Type Culture Collection (ATCC, Manassas, USA). The MCF7, T47D, ZR-75-1, SKBR3, JIMT-1 and KPL4 cell lines were cultured in RPMI 1640 medium (Sigma, St. Louis, MO, USA), while the BT474, MDA-MB231 and MDA-MB361 lines were cultured in DMEM medium (Sigma). All culture media were supplemented with 10% fetal bovine serum (FBS) (Sigma), an antibiotic-antimycotic solution (1X) (Sigma) and L-glutamine (2 mM) (Invitrogen GmbH, Karslruhe, Germany). The cultures were maintained in an incubator at 37°C and 5% CO_2_.

### Tumor samples for primary culture

The study on primary cultures was approved by the ethics institutional review board for "Biobanking and use of human tissue for experimental studies" of the Pathology Units of the Azienda Ospedaliera Città della Salute e della Scienza di Torino. At our Institution, written informed consent is obtained from patients for the use of residual tissues from the diagnostic procedures in research studies.

We analyzed the cells of a triple negative breast carcinoma (TNBC) that metastasized to the peritoneum, giving rise to a peritoneal effusion. The triple negative phenotype was confirmed by immunohistochemistry (IHC) for the estrogen receptor (ER) (Clone SP1, 1:50 diluted, Cell Marque, Rocklin, California), progesterone receptor (PR) (Clone 1A6, 1:50 diluted, Leica Biosystems, Newcastle Upon Tyne, United Kingdom) and by FISH for the *HER2* gene on a cell block obtained after centrifugation of an aliquot of the effusion. The remaining part was used to set up a short-term primary culture according to a protocol recently described [15]. The epithelial origin of the cells was confirmed by the positive expression of cytokeratins (clones AE1/AE3 and PCK26, pre-diluted, Ventana-Diapath, Tucson, AZ, USA) and by the absence of the mesothelial marker calretinin (polyclonal; 1:100 diluted, Invitrogen) using an immunohistochemical procedure on cells grown directly on sterilized slides [15].

### G-Banding and karyotyping

Metaphases for performing conventional and molecular cytogenetic analysis (M-FISH and FISH) were obtained by using standardized harvesting protocols, as recently described [16].

Metaphases image acquisition and subsequent karyotyping were performed by using a Nikon microscope with the cytogenetic software CytoVision System (Applied Imaging, Santa Clara, CA). Between 10 and 26 metaphase cells with good dispersion and morphology were analyzed for each cell line. Chromosome aberrations were described according to the International System for Human Cytogenetic Nomenclature 2013 (ISCN) [17].

### Multi-color fluorescence *in situ*hybridization (M-FISH)

M-FISH was performed as recently described [16]. Briefly, we used a probe cocktail containing 24 differentially labeled chromosome-specific painting probes (24xCyte kit MetaSystems, Altlussheim, Germany) that was denatured and hybridized to denatured tumor metaphase chromosomes. The slides were incubated at 70°C in saline solution (2xSSC), denatured in NaOH, dehydrated in an ethanol series, air-dried, covered with 10 μl of the probe cocktail (denatured) and finally hybridized for two days at 37°C. Subsequently, the slides were washed with post-hybridization buffers, dehydrated in an ethanol series and counter-stained with 10 μl of DAPI/antifade. The Metafer system and the Metasytems ISIS software (Carl Zeiss, Metasystems, GmbH) were used for signal detection and metaphase analysis. At least 10 metaphases exhibiting the same derivative chromosomes were studied for each cell line.

### FISH for the *HER2, STARD3*and *TOP2A*genes

FISH experiments were performed to define the *HER2, STARD3* (17q12) and *TOP2A* (17q21-q22) gene status and mapping. In *HER2* amplified tumors *STARD3* is included in the smallest region of amplification (SRA) involving *HER2*, whereas *TOP2A* is reported to pertain to a separate amplicon.

Two commercial dual-color probes for *HER2* (SpectrumOrange)/CEP17 (SpectrumGreen) and *TOP2A* (SpectrumOrange)/CEP17 (SpectrumGreen) (all from Abbott Molecular, Downers Grove, IL, USA) were used separately on each cell line.

For the *STARD3* gene, FISH studies were performed using both an alpha satellite probe specific for Chr17 (CEP17) that was directly labeled with a green fluorochrome (Abbott molecular) and a *STARD3* specific locus probe fosmid WI2-2398I17 (17q12) that was made in-house. The clone was obtained from BACPAC Resources Center (Children’s Hospital Oakland Research Institute, CA, USA). The UCSC database (http://genome.ucsc.edu, February 2009 release) was queried to localize the probe. The fosmid was expanded, extracted using the QIAGEN Plasmid Purification Kit (QiagenGmbH, Hilden, Germany) and then directly labeled with SpectrumOrange-dUTP (Abbott Molecular), using the Nick Translation Kit (Abbott Molecular) according to the manufacturer’s instructions. The fosmid clone was tested on metaphase and interphase cells of healthy donors, obtained using conventional cytogenetic methods, to analyze the position and strength of the signal, the presence/absence of background and cross-hybridization and the hybridization efficiency.

FISH with the *HER2*/CEP17, *STARD3*/CEP17 and *TOP2A*/CEP17 probes was performed separately on each cell line on fresh slides from methanol and acetic acid fixed cells according to the manufacturers’ instructions. Briefly, the slides were washed at 37°C in 2x saline-sodium citrate buffer (SSC), dehydrated in an ethanol series, air-dried, covered with 10 μl of probe, co-denatured in HYBrite System at 70°C for 5 min and hybridized overnight at 37°C. Slides were then washed with a post-hybridization buffer (2xSSC/0.3% Nonidet P-40), dehydrated in an ethanol series and counter-stained with 10 μl DAPI/antifade. Metaphases and nuclei were selected with an AxioImager Z1 epifluorescence microscope (Carl Zeiss, Germany). Analysis of the signal pattern on the interphase nuclei and metaphases was performed with the ISIS software. The number of FISH signals and the localization of the signals were analyzed in at least 10 metaphases and interphase nuclei.

## Results

### Structural alterations of Chr17

**Figure 1:**
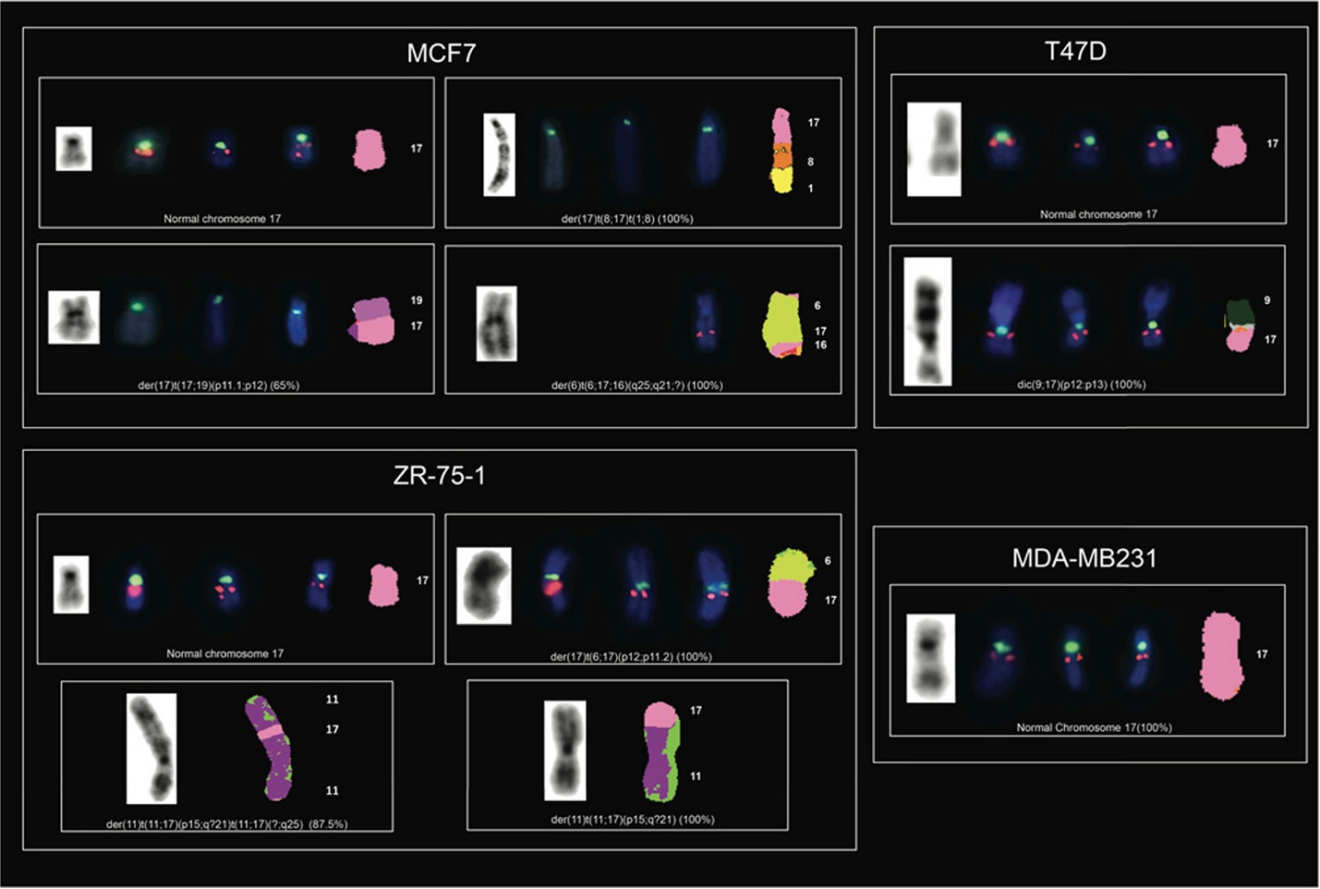
**Analysis of Chr17 using G-Banding, dual-color FISH (** ***HER2*** **/CEP17** ***, STARD3*** **/CEP17** **and** ***TOP2A*** **/CEP17) and M-FISH in the MCF7, T47D, ZR-75-1 and MDA-MB231 not** ***HER2*** **amplified breast cancer cell lines.** Rearranged chromosomes containing a portion of Chr17 are visualized by G-Banding technique on the left and by M-FISH on the right. For M-FISH the classified color of Chr17 is shown in pink, the translocation partners are numbered on the right hand side of the chromosomes and the frequency at which each abnormality was observed is indicated in brackets at the end of each abnormality. CEP17, *HER2, STARD3* and *TOP2A* are shown in the middle by dual-color FISH (*HER2*/CEP17, *STARD3*/CEP17, *TOP2A*/CEP17, respectively) whenever mapped to the corresponding derivatives (CEP17 is green-labeled; *HER2, STARD3* and *TOP2A* genes are red-labeled).

**Figure 2:**
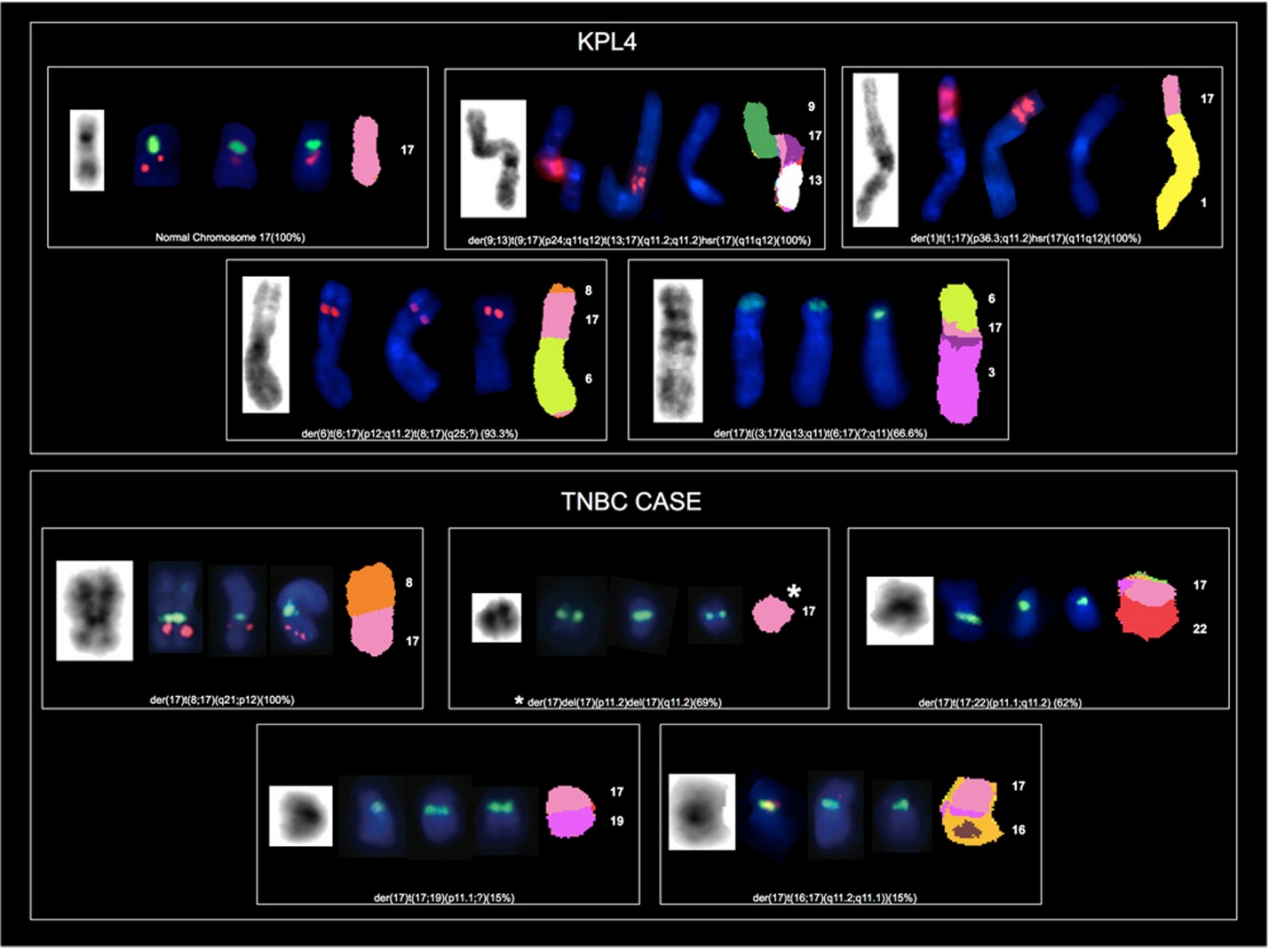
**Analysis of Chr17 using G-Banding, dual-color FISH (** ***HER2*** **/CEP17** ***, STARD3*** **/CEP17 and** ***TOP2A*** **/CEP17) and M-FISH in KPL4** ***HER2*** **amplified breast cancer cell line showing four translocated Chr17 in addition to the normal-appearing copies of Chr17 and in one triple negative breast cancer case (TNBC) showing five rearranged copies of Chr17.** Rearranged chromosomes containing a portion of Chr17 are visualized by G-Banding technique on the left and by M-FISH on the right. For M-FISH the classified color of Chr17 is shown in pink, the translocation partners are numbered on the right hand side of the chromosomes and the frequency at which each abnormality was observed is indicated in brackets at the end of each abnormality. CEP17, *HER2, STARD3* and *TOP2A* are shown in the middle by dual-color FISH (*HER2*/CEP17, *STARD3*/CEP17, *TOP2A*/CEP17, respectively) whenever mapped to the corresponding derivatives (CEP17 is green-labeled; *HER2, STARD3* and *TOP2A* genes are red-labeled). In the TNBC cells the chromosome in which we identified Chr17 material only is a der(17)del(17)(p11.2)del(17)(q11.2) with a deletion on both short and long arm involving 17q12-q21.

**Figure 3:**
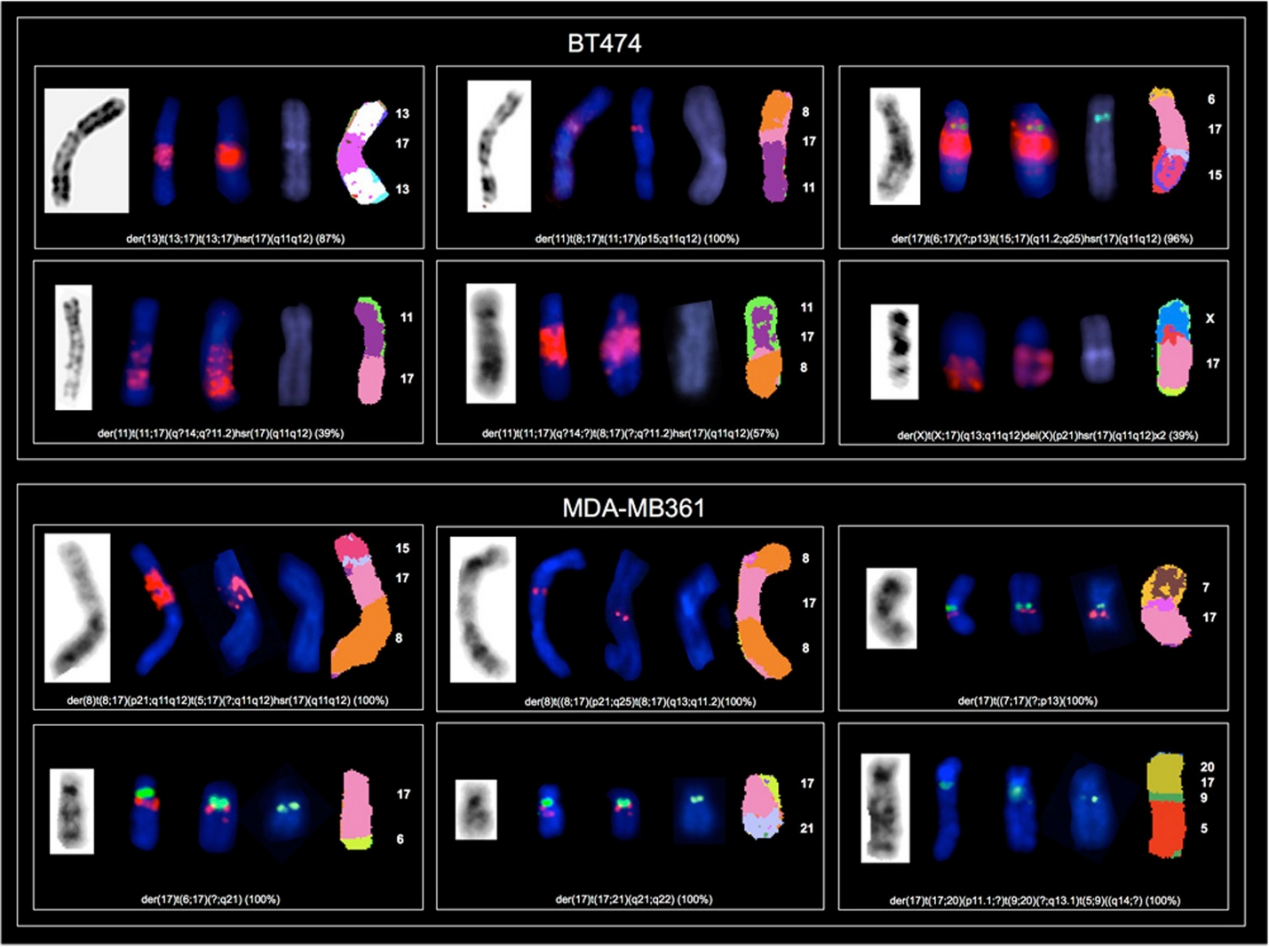
**Analysis of Chr17 using G-Banding, dual-color FISH (** ***HER2*** **/CEP17** ***, STARD3*** **/CEP17** **and** ***TOP2A*** **/CEP17) and M-FISH in BT474 and MDA-MB361** ***HER2*** **amplified breast cancer cell lines showing six translocated copies of Chr17.** Rearranged chromosomes containing a portion of Chr17 are visualized by G-Banding technique on the left and by M-FISH on the right. For M-FISH the classified color of Chr17 is shown in pink, the translocation partners are numbered on the right hand side of the chromosomes and the frequency at which each abnormality was observed is indicated in brackets at the end of each abnormality. CEP17, *HER2, STARD3* and *TOP2A* are shown in the middle by dual-color FISH (*HER2*/CEP17, *STARD3*/CEP17, *TOP2A*/CEP17, respectively) whenever mapped to the corresponding derivatives (CEP17 is green-labeled; *HER2, STARD3* and *TOP2A* genes are red-labeled).

**Figure 4:**
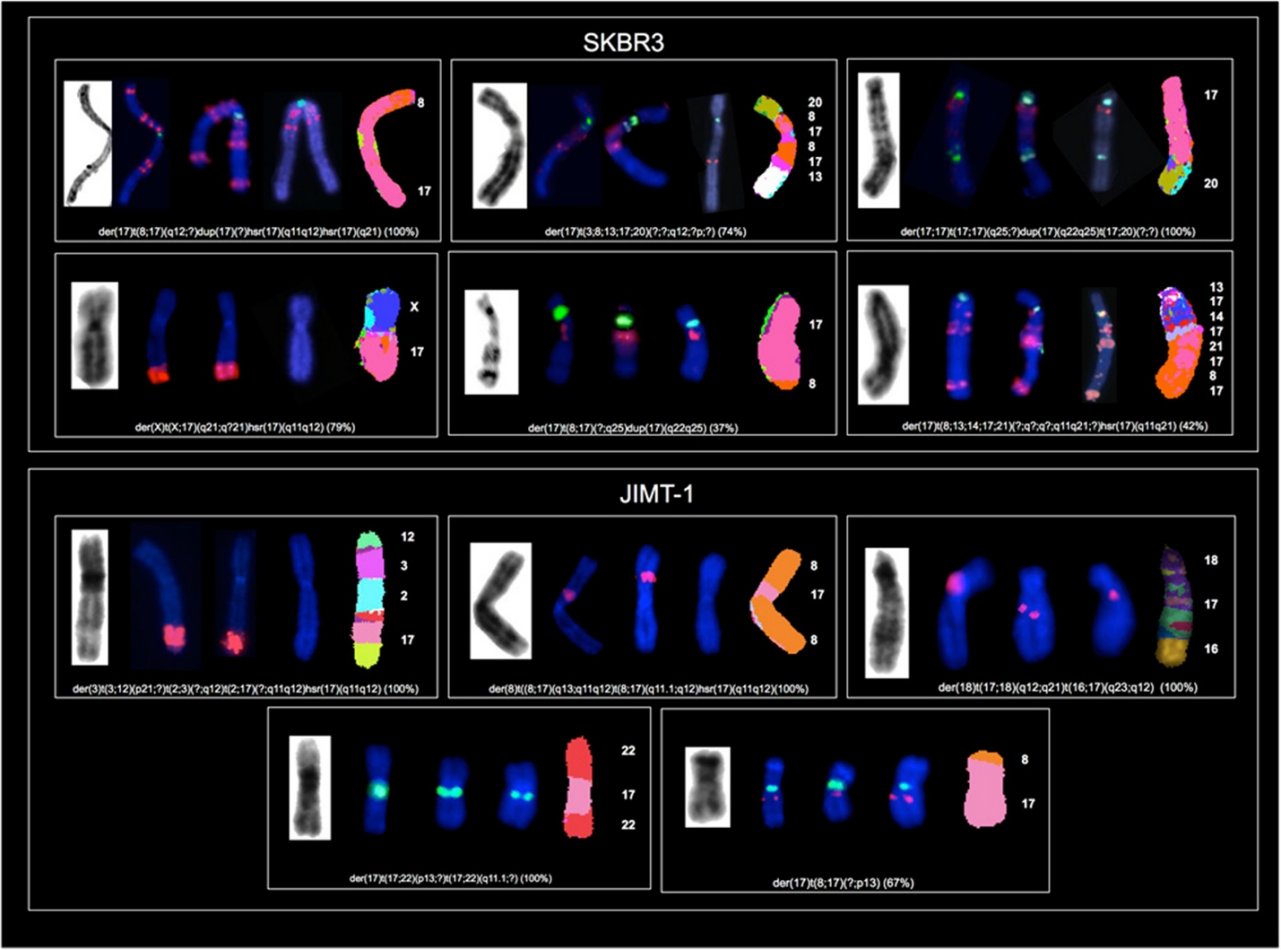
**Analysis of Chr17 using G-Banding, dual-color FISH (** ***HER2*** **/CEP17** ***, STARD3*** **/CEP17** **and** ***TOP2A*** **/CEP17) and M-FISH in SKBR3 and JIMT-1** ***HER2*** **amplified breast cancer cell lines showing four or six translocated copies of Chr17.** Rearranged chromosomes containing a portion of Chr17 are visualized by G-Banding technique on the left and by M-FISH on the right. For M-FISH the classified color of Chr17 is shown in pink, the translocation partners are numbered on the right hand side of the chromosomes and the frequency at which each abnormality was observed is indicated in brackets at the end of each abnormality. CEP17, *HER2, STARD3* and *TOP2A* are shown in the middle by dual-color FISH (*HER2*/CEP17, *STARD3*/CEP17, *TOP2A*/CEP17, respectively) whenever mapped to the corresponding derivatives (CEP17 is green-labeled; *HER2, STARD3* and *TOP2A* genes are red-labeled).

The specific Chr17 alterations we found are detailed in Table 1. In 8 out of the 9 cell lines analyzed we identified 39 rearranged chromosomes containing a portion of Chr17 (mainly its long arm) (Figures 1, 2, 3 and 4). The triple negative MDA-MB231 cells showed no Chr17 alterations, while the *HER2* amplified MDA-MB361, SKBR3 and JIMT-1 cell lines carried no normal copies of Chr17. In particular, the SKBR3 cells harbored 10 different types of structural abnormalities on Chr17, making it the cell line with the highest frequency of structural abnormalities. The lowest frequency of Chr17 abnormalities was observed in HER2 negative cells, which carried between 2 and 3 different types of alterations.

**Table 1:** **Aberrations of Chr17 as revealed by G-Banding, M-FISH and FISH in nine breast cancer cell lines and a primary culture raised from a triple negative breast carcinoma**

Cell lines	Type of rearrangement
**MCF7 (ER+/HER2-)**	der(6)t(6;17;16)(q25;q21;?)[100],der(17)t(8;17)t(1;8)[100],der(17)t(17;19)(p11.1;p12)[65]
**T47D (ER+/HER2-)**	dic(9;17)t(9;17)(p12;p13)[100]
**ZR-75-1 (ER+/HER2-)**	der(11)t(11;17)(p15;q?21)[100],der(11)t(11;17)(p15;q?21)t(11;17)(?;q25)[88],der(17)t(6;17)(p12;p11.2)[100]
**BT474 (ER+/HER2+)**	der(X)t(X;17)(q13;q11q12)del(X)(p21)hsr(17)(q11q12)x2[39],der(11)t(8;17)(q21.1;q11q12)t(11;17)(p15;q11q12)x2[100],der(11)t(11;17)(q?14;q?11.2)hsr(17)(q11q12)[39],der(11)t(11;17)(q?14;?)t(8;17)(?;q?11.2)hsr(17)(q11q12)x2[57],der(13)t(13;17)(q10;q11q12)t(13;17)(q10;q11q12)hsr(17)(q11q12)x2[87],der(17)t(6;17)(?;p13)t(15;17)(q11.2;q25)hsr(17)(q11q12)x2[96]
**MDA-MB361 (ER+/HER2+)**	der(8)t(8;17)(p21;q11q12)t(5;17)(?;q11q12)hsr(17)(q11q12)[100],der(8)t(8;17)(p21;q25)t(8;17)(q13;q11.2)[100],der(17)t(6;17)(?;q21)[100],der(17)t(7;17)(?;p13)[100], der(17)t(17;20)(p11.1;?)t(9;20)(?;q13.1)t(5;9)(q14;?)[100], der(17)t(17;21)(q21;q22)[100]
**SKBR3 (ER-/HER2+)**	der(X)t(X;17)(q21;q?21)hsr(17)(q11q12)x2[79], der(17)t(8;17)(q12;?)dup(17)(?)hsr(17)(q11q12)hsr(17)(q21)[100],der(17)t(8;17)(?;q25)dup(17)(q22q25)[37],der(17)t(8;13;14;17;21)(?;q?;q?;q11q12;?)hsr(17)(q11q21) [42],der(17)t(3;8;13;17;17;20)(?;?;q12;q12;?p;?)[74], der(17;17)t(17;17)(q25;?)dup(17)(q22q25)t(17;20)(?;?)[100]
**JIMT-1 (ER-/HER2+)**	der(3)t(3;12)(p21;?)t(2;3)(?;q12)t(2;17)(?;q11q12)hsr(17)(q11q12)[100], ,der(8)t(8;17)(q13;q11q12)t(8;17)(q11.1;q12)hsr(17)(q11q12)[100],der(17)t(8;17)(?;p13)[67],der(17)t(17;22)(p13;?)t(17;22)(q11.1;?)[100],der(18)t(17;18)(q12;q21)t(16;17)(q23;q12)[100]
**KPL4 (ER-/HER2+)**	der(1)t(1;17)(p36.3;q11q12)hsr(17)(q11q12)[100],der(6)t(6;17)(p12;q11.2)t(8;17)(q25;?)[93],der(9;13)t(9;17)(p24;q11q12)t(13;17)(p11.2;q11.2)hsr(17)(q11q12)[100], der(17)t(3;17)(q13;q11)t(6;17)(?;q11)[66.6]
**MDA-MB231 (ER-/HER2-)**	//
**TNBC CASE (ER-/HER2-)**	der(17)t(8;17)(q21;p12)[100],der(17)t(16;17)(q11.2;q11.1)[15],der(17)del(17)(p11.2)del(17)(q11.2)[69],der(17)t(17;19)(p11.1;?)[15],der(17)t(17;22)(p11.1;q11.2)[62]

The % of cells for which each abnormality was observed is indicated at the end of each abnormality within square brackets. The number of cells examined for chromosome count was 26 for MCF7 cells; 24 for T47D cells; 10 for ZR-75-1 cells and for BT474 cells; 10 for MDA-MB361 cells; 19 for SKBR3 cell; 18 for JIMT-1 cells; 15 for KPL4 cells, 14 for MDA-MB231 cells and 13 for the triple negative breast cancer case (TNBC).

We defined nine regions of Chr17 frequently involved in the observed structural alterations: 17p11, 17p13, 17q11.2, 17q11-12, 17q12, 17q21, 17q22, 17q23 and 17q25. The 17q11-12 region was the most frequent long arm portion involved in structural alterations. This region was affected in the BT474, MDA-MB361, SKBR3, JIMT-1 and KPL4 *HER2* amplified cell lines, while 17p11 and 17p13 were commonly affected in the MCF7, ZR-75-1, MDA-MB361 and the SKBR3 and in T47D, MDA-MB361, JIMT-1 cells, respectively (Table 1).

Using G-Banding, numerous complex derivative chromosomes containing material from Chr17 were observed in all cell lines except for MDA-MB231. Some of the derivative chromosomes were present in duplicate (Table 1). Chr17 deletions and dicentric chromosomes were observed only in the T47D and SKBR3 cells.

M-FISH demonstrated that chromosome 8 and chromosome 11 were the most frequent translocation partners of Chr17 (Table 2). Twelve different rearrangements between Chr17 and chromosome 8, involving mainly their long arms (8q11.1, 8q12, 8q13, 8q21 and 8q24) were identified in MCF7, MDA-MB361, BT474, SKBR3 and JIMT-1 cells. Similarly, 5 translocations between Chr17 (long arm) and chromosome 11 (involving 11p15, 11q13 and 11q23) were identified in ZR-75-1 and BT474 cells. Translocations with chromosome 6 were observed in five cell lines, and translocations between Chr17 and chromosomes X, 1, 3, 7 and 16 were observed only in HER2 positive cells (Table 2). We identified 5 different alterations of Chr17 in the primary TNBC culture, involving both the short (17p11.1, 17p11.2, 17p12) and the long (17q11.1 and 17q11.2) arms. In addition, numerous complex Chr17 derivatives containing material from chromosomes 8, 16, 19 and 22 were observed.

**Table 2:** **Frequency of translocation partners of Chr17 in nine breast cancer cell lines**

Translocation partner	Chromosomal abnormality	Number of abnormalities	No of cell lines	Cell lines
Chromosome 8	der	12	5	MCF7, MDA-MB361, SKBR3, JIMT-1, BT474
Chromosome 11	der	5	2	ZR-75-1, BT474
Chromosome 6	der	5	5	MCF7, ZR-75-1, MDA-MB361, BT474, KPL4
Chromosome X	der	2	2	BT474, SKBR3
Chromosome 9	der	2	2	KPL4, T47D
Chromosome 9	dic	1	1	T47D
Chromosome 3	der	2	2	JIMT-1, KPL4
Chromosome 7	der	1	1	MDA-MB361
Chromosome 13	der	2	2	BT474, KPL4
Chromosome 1	der	1	1	KPL4
Chromosome 16	der	1	1	JIMT-1
Chromosome 17	der	1	1	SKBR3
Chromosome 19	der	1	1	MCF7
Chromosome 20	der	1	1	MDA-MB361
Chromosome 21	der	1	1	MDA-MB361
Chromosome 22	der	1	1	JIMT-1

der = derivative chromosome; dic = dicentric chromosome.

### Mapping CEP17 and the 17q12–q21 amplicon

We considered the chromosomal correlation of *HER2, STARD3* and *TOP2A* genes mapping to 17q12–q21 with CEP17 as shown by FISH on metaphase chromosomes and we compared the results to the interphase pattern. By M-FISH we reported the specific rearrangements. Out of the 39 rearranged chromosomes containing a portion of Chr17 identified by M-FISH, 12 harbored *HER2*, *STARD3* (which mapped always together) and *TOP2A*; 16 harbored *HER2* and *STARD3*, 1 harbored only *TOP2A*, 2 did not show either CEP17, *HER2*, *STARD3* or *TOP2A* signals.

Notably, 8 of the 39 rearranged chromosomes carried CEP17 signals without *HER2* and *STARD3* signals and 14 harbored *HER2* and *STARD3* genes but not CEP17.

The specific patterns observed by FISH in each cell line are reported below.

#### Triple negative cell lines

**Figure 5:**
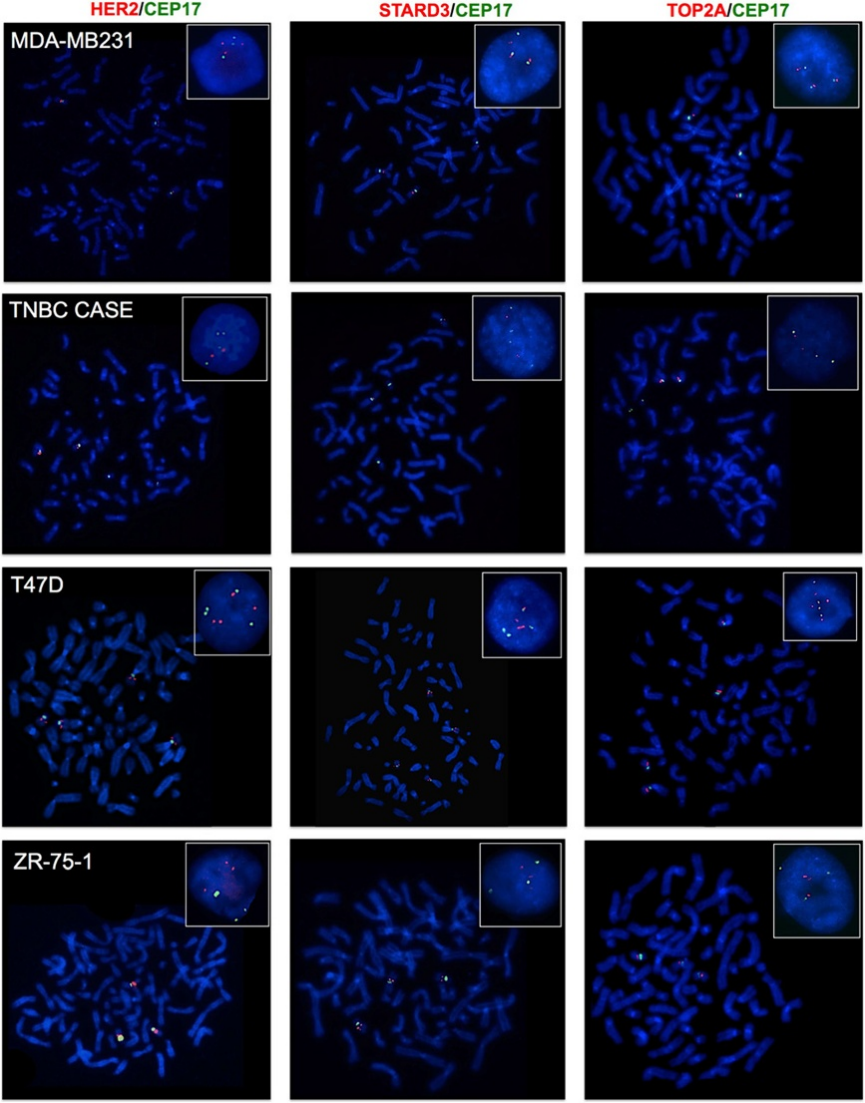
**Representative FISH images of the MDA-MB231, T47D and ZR-75-1 breast cancer cells and one TNBC case using** ***HER2*** **/CEP17,** ***STARD3*** **/CEP17 and** ***TOP2A*** **/CEP17 dual-color probes.** Metaphase spreads are shown and boxes indicate representative interphase nuclei for each case. None of these cell lines showed amplification of the *HER2, STARD3* or *TOP2A* genes. Gene signals are red-labeled, CEP17 signals are green-labeled.

In the MDA-MB231 triple negative cells the FISH (both in interphase and metaphase) and M-FISH patterns corresponded to three copies of normal Chr17, each with one CEP17 green signal and one red signal corresponding to either *HER2, STARD3* or *TOP2A* (Table 3, Figures 5 and 1).

**Table 3:** ***HER2*** **and** ***STARD3*** **FISH pattern and complex Chr17 rearrangements in nine breast cancer cell lines and one primary culture raised from a triple negative breast carcinoma**

Cell line	CEP 17 signals (green)	***HER2***signals (red)		Chr17		Complex abnormalities encompassing ***HER2***Amplification
		Cluster	Individual	Normal	Derivatives	
MCF7	4	0	2	2*	der(17)t(8;17)t(1;8)	
					der(17)t(17;19)(p11.1;p12)	
T47D	4	0	4	2*	dic(9;17)(p12;p13)*x2	
ZR-75-1	3	0	3	2*	der(17)t(6;17)(p12;p11.2)*	
BT474	6	9	6	4*	der(17)t(6;17)(?;p13)t(15;17)(q11.2;q25)hsr(17)(q11q12**)x2	der(X)t(X;17)(q13;q11q12)del(X)(p21)hsr(17)(q11q12**)x2
						der(11)t(8;17)(q21.1;q11q12*)t(11;17)(p15;q11q12)x2
						der(11)t(11;17)(q?14;q?11.2)hsr(17)(q11q12**)
						der(11)t(11;17)(q?14;?)t(8;17)(?;q?11.2)hsr(17)(q11q12**)x2
						der(13)t(13;17)(q10;q11q12)t(13;17)(q10;q11q12)hsr(17)(q11q12**)x2
						der(17)t(6;17)(?;p13)t(15;17)(q11.2;q25)hsr(17)(q11q12**)x2
MDA-MB361	4	1	4	0	der(17)t(6;17)(?;q21)*	der(8)t(8;17)(p21;q11q12)t(5;17)(?;q11q12)hsr(17)(q11q12**)
					der(17)t(7;17)(?;p13)*	der(8)t(8;17)(p21;q25)t(8;17)(q13;q11.2*)
					der(17)t(17;20)(p11.1;?)t(9;20)(?;q13.1)t(5;9)(q14;?)	
					der(17)t(17;21)(q21;q22)*	
SKBR3	7	16	4	0	der(17)t(8;17)(q12;?)dup(17)(?)hsr(17)(q11q12**^/^**^/^**^/^**^/^**^/^**) hsr(17)(q21)x2	der(X)t(X;17)(q21;q?21)hsr(17)(q11q12**)x2
					der(17)t(8;17)(?;q25)dup(17)(q22q25)*	der(17)t(8;17)(q12;?)dup(17)(?)hsr(17)(q11q12**^/^**^/^**^/^**^/^**^/^**)hsr(17)(q21)x2
					der(17)t(8;13;14;17;21)(?;q?;q?;q11q12;?) hsr(17)(q11q21**^/^**)	der(17)t(8;13;14;17;21)(?;q?;q?;q11q12;?)hsr(17)(q11q21**^/^**)
					der(17)t(3;8;13;17;17;20)(?;?;q12*;q12*;?p;?)	
					der(17;17)t(17;17)(q25;?)dup(17)(q22q25)t(17;20)(?;?)*	
JIMT-1	2	2	2	0	der(17)t(8;17)(?;p13)*	der(3)t(3;12)(p21;?)t(2;3)(?;q12)t(2;17)(?;q11q12)hsr(17)(q11q12**)
					der(17)t(17;22)(p13;?)t(17;22)(q11.1;?)	der(8)t(8;17)(q13;q11q12)t(8;17)(q11.1;q12)hsr(17)(q11q12**)
KPL4	3	2	3	2*	der(6)t(6;17)(p12;q11.2*)t(8;17)(q25;?)	der(1)t(1;17)(p36.3;q11q12)hsr(17)(q11q12**)
					der(17)t(3;17)(q13;q11)t(6;17)(?;q11)	der(9;13)t(9;17)(p24;q11q12)t(13;17)(p11.2;q11.2)hsr(17)(q11q12**)
MDA-MB231	3	0	3	3*	0	0
TNBC CASE	4	0	2	0	der(17)t(8;17)(q21;p12)*x2	
					der(17)del(17)(p11.2)del(17)(q11.2)	
					der(17)t(17;22)(p11.1;q11.2)	

*Indicates the presence of one red signal (*HER2*) on a normal Chr17 or on a derivative Chr17.

**Indicates the presence of one red cluster (*HER2*) on a derivative Chr17.

**^/^**Indicates the presence of two red clusters (*HER2*) on a derivative Chr17.

**^/^**^/^**^/^**^/^**^/^**Indicates the presence of six red clusters (*HER2*) on a derivative Chr17.

Scoring of interphase nuclei to obtain the final result on *HER2* gene status performed based both on a dual-FISH and a single FISH assay (according o the new ASCO/CAP guidelines [19]) showed no differences in the final result for each of the cell lines.

The TNBC primary culture nuclei displayed the same FISH pattern for the *HER2, STARD3*, *TOP2A* genes and CEP17. Four green CEP17 signals and two red signals were observed (Figures 5 and 2). Two red and two green signals corresponded to two Chr17 derivatives, namely der(17)t(8;17)(q21;p12)x2 (100%), while the other two green signals (without the *HER2, STARD3* and *TOP2A* genes) mapped to der(17)t(17;22)(p11.1;q11.2) (62%) and der(17)del(17)(p11.2)del(17)(q11.2) (69%). This last Chr17 derivative showed deletion on both short and long arms involving the 17q12-21 region (Figure 2).

#### ER+/HER2 not amplified cell lines

In T47D and ZR-75-1 interphase nuclei, the same copy numbers of *HER2, STARD3* and *TOP2A* genes and of CEP17 were observed (Table 3, Figure 5). Four copies were observed in the T47D nuclei and three in the ZR-75-1 nuclei (Table 3, Figure 5).

The T47D cells showed two normal Chr17 and two Chr17 derivatives carrying both CEP17 and the three genes (Figures 5 and 1). M-FISH showed that the derivative chromosome previously reported as der(9)t(9;17)(p13;q11) [18] was a dic(9;17)t(9;17)(p12;p13) (Figure 1).

In ZR-75-1, M-FISH showed that *HER2, STARD3* and *TOP2A* genes mapped to two normal Chr17 and one derivative Chr17 (Table 3, Figures 5 and 1).

**Figure 6:**
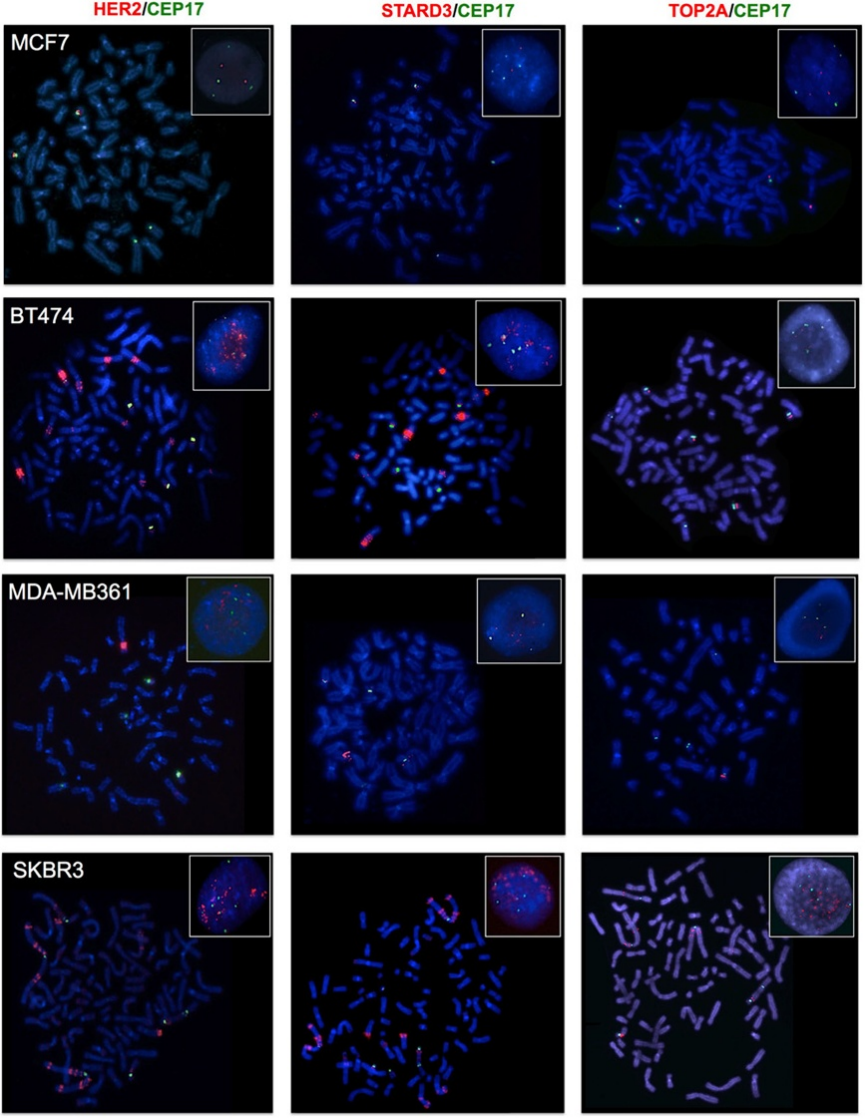
**Representative FISH images of the MCF7, BT474, MDA-MB361 and SKBR3 breast cancer cell lines using** ***HER2*** **/CEP17,** ***STARD3*** **/CEP17 and** ***TOP2A*** **/CEP17 dual-color probes.** Metaphase spreads are shown and boxes indicate representative interphase nuclei for each case. FISH images for the BT474, MDA-MB361 and SKBR3 cells demonstrated *HER2* and *STARD3* gene amplification, while *TOP2A* gene amplification was observed in the SKBR3 cells only. Gene signals are red-labeled, CEP17 signals are green-labeled.

MCF7 interphase nuclei displayed four CEP17 green signals and two red signals for the *HER2* and *STARD3* genes (Table 3, Figure 6). This pattern corresponded to one CEP17 signal and one copy of the *HER2* and *STARD3* genes located on two normal Chr17 and two CEP17 signals on two Chr17 derivatives as confirmed by M-FISH (Figure 1). The FISH pattern for *TOP2A* was similar to that observed for the *HER2* and *STARD3* genes, with the only exception of having an additional *TOP2A* copy mapping on a derivative chromosome 6 (Figures 6 and 1).

#### HER2 amplified cell lines

*HER2, TOP2A* and *STARD3* gene amplifications were found within chromosomes as homogeneously staining regions (HSRs) but not in extra-chromosomal, double-minute chromosomes (DMs). All of these cell lines showed *HER2* and *STARD3* co-amplification.

In BT474 interphase nuclei, six CEP17 signals and several clusters of *HER2* and *STARD3* were observed. This pattern corresponded to nine clusters and six individual red signals in metaphases (Figure 6). By comparing FISH and M-FISH data, we showed that four CEP17 and four red signals were located on four normal copies of Chr17, and two CEP17 signals and two clusters of red signals on two Chr17 derivatives as shown by M-FISH: der(17)t(6;17)(?;p13)t(15;17)(q11.2;q25)hsr(17)(q11q12)x2 (96%). The remaining seven clusters of red signals mapped to five previously unreported highly rearranged chromosomes (Table 3, Figure 3).

BT474 cells showed normal *TOP2A* gene copy numbers, and four red signals were observed on four normal copies of Chr17 only (Figures 6 and 3).

In the MDA-MB361 nuclei four CEP17 signals, one red cluster and four individual red signals (*HER2* and *STARD3*) were observed (Figure 6). None of these green and red signals were located on normal copies of Chr17 (Table 3, Figure 3). Three individual red signals were correlated with the centromeric locus and located on three Chr17 derivatives. The other individual red signal mapped to a chromosome 8 derivative and the only red cluster, indicative of *HER2* and *STARD3* amplification, was located on another chromosome 8 derivative. The remaining CEP17 signals, without red signal (*HER2* and *STARD3* deletion), mapped to a complex translocation of Chr17 involving chromosomes 5, 9 and 20 (Figure 3).

These cells harbored a *TOP2A* deletion, as four chromosomes with CEP17 were identified, but only one of them had a *TOP2A* signal (Figures 6 and 3).

In the SKBR3 cells, *HER2* and *STARD3* co-amplification was observed in 100% of metaphase and interphase nuclei analyzed. Seven CEP17 signals and sixteen clusters and four individual red signals (*HER2* and *STARD3*) were observed on numerous highly rearranged chromosomes (Table 3 and Figure 6). In particular, two CEP17 and one red signal mapped to the dicentric Chr17, der(17;17)t(17;17)(q25;?)dup(17)(q22q25)t(17;20)(?;?) (100%), which had not been previously reported (Figure 4). In two Chr17 derivatives *TOP2A* was co-amplified with *HER2* either as a single amplicon (der(17)t(8;13;14;17;21)(?;q?;q?;q11q12;?)hsr(17)(q11q21)) or as separate amplicons (der(17)t(8;17)(q12;?)dup(17)(?)hsr(17)(q11q12)hsr(17)(q21)) (Figure 4). In addition, *TOP2A* deletion was detected on der(X)t(X;17)(q21;q?21)hsr(17)(q11q12). In the remaining derivative chromosomes without gene amplification, *TOP2A* showed the same FISH pattern observed for *HER2*, in which all distinct *HER2* genes were accompanied by distinct *TOP2A* genes (Figures 4 and 6).

**Figure 7:**
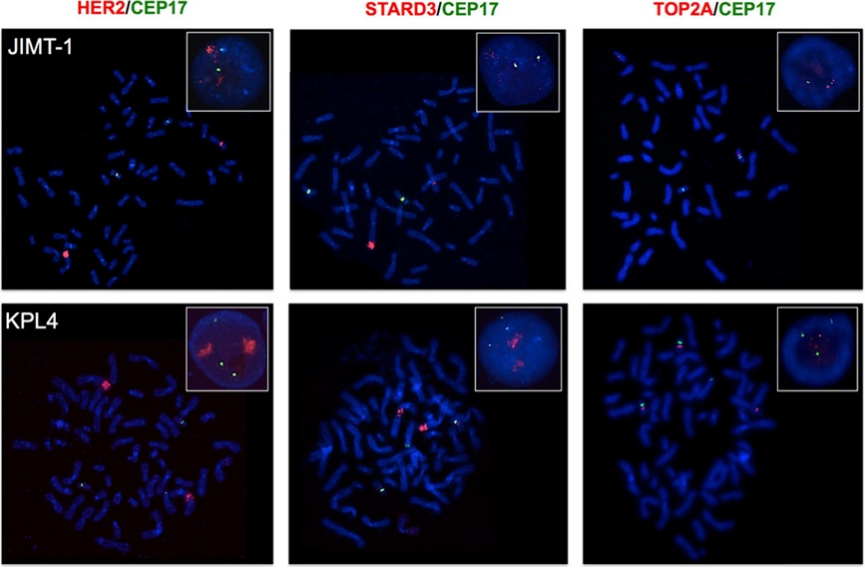
**Representative FISH images of the JIMT-1 and KPL4 breast cancer cell lines using** ***HER2*** **/CEP17,** ***STARD3*** **/CEP17 and** ***TOP2A*** **/CEP17 dual-color probes.** Metaphase spreads are shown and boxes indicate representative interphase nuclei for each case. FISH images for the JIMT-1 and KPL4 cells demonstrate *HER2* and *STARD3* gene amplification, while *TOP2A* gene amplification was not observed in these cells. Gene signals are red-labeled, CEP17 signals are green-labeled.

In the JIMT-1 cells, two CEP 17 signals and two clusters and two individual red signals were observed for *HER2* and *STARD3* genes (Figure 7). The two clusters of red signals mapped to two chromosomes lacking CEP17 (Table 3 and Figure 4). One of the two individual red signals was observed on a Chr17 derivative while the other was on a chromosome 18 derivative (Table 3 and Figure 4). We also observed *HER2* and *STARD3* deletion on der(17)t(17;22)(p13;?)t(17;22)(q11.1;?) (Figure 4).

*TOP2A* was not amplified and the FISH pattern showed two red and two CEP17 signals: one red signal mapped to a Chr17 derivative, while the other mapped to a chromosome 18 derivative. In addition, a loss of the *TOP2A* gene (*TOP2A* deletion) was observed on der(17)t(17;22)(p13;?)t(17;22)(q11.1;?), similar to that observed for the *HER2* and *STARD3* genes (Figure 4). *TOP2A* signals were not observed on derivative chromosomes with *HER2* amplification (Figure 4).

The KPL4 cells showed three CEP17 signals and two clusters and three individual red signals of *HER2* and *STARD3* genes (Figure 7). Two CEP17 and two red signals were located on two normal copies of Chr17, the other green and red individual signals corresponded to complex rearrangements involving Chr17 (Table 3, Figure 2). Like the JIMT-1 cells, the *HER2* and *STARD3* gene clusters were located on highly rearranged chromosomes (Table 3, Figure 2).

These cells did not show *TOP2A* gene amplification (Figure 7). Instead, one CEP17 signal and one red signal were observed each on two distinct normal Chr17 copies, and one red signal mapped to a chromosome 6 derivative (Figure 2).

## Discussion

By comparing the *HER2*/CEP17 FISH pattern in metaphase *versus* interphase nuclei, the present study demonstrated that a CEP17 signal rarely corresponds to a single intact Chr17, in both HER2+ and HER2- cell lines. It is well known that cells of long-term cultures may show high chromosomal rearrangements, however Chr17 was altered even in the short-term TNBC primary culture. Although obtained in a single primary cell line this specific finding may corroborate our hypothesis that chromosomal alterations involving Chr17 in breast cancer may be indeed very complex and merit further investigation in primary cell cultures obtained from carcinomas of different phenotypes.

This extensive cytogenetic analysis of Chr17 demonstrated that only the MDA-MB231 triple negative showed true polysomy (normal chromosome acquisition) as the only Chr17 alteration. The BT474 HER2-positive cells showed Chr17 polysomy together with different Chr17 rearrangements. In ER+/*HER2* not amplified cell lines normal copies of Chr17 coexist with rearranged Chr17 copies that either harbor or do not harbor the *HER2* gene. On the other hand, some of the *HER2* amplified cell lines did not show any normal copies of Chr17 and the *HER2*-*STARD3* gene clusters were observed as HSR on complex rearranged chromosomes. In addition, the Chr17 derivatives (carrying CEP17) did not always show *HER2* gene clusters, and these latter were not exclusively observed in Chr17 derivatives. In particular, in the BT474 cells 7 of 9 *HER2* gene clusters were found on derivatives lacking CEP17.

In breast cancer specimens the analysis of the *HER2* gene in interphase nuclei is requested after an equivocal immunohistochemical result (score 2+) of HER2 expression [8, 19]. Either double-signal (*HER2* and CEP17 probes) or single-signal (*HER2* probe) assays may be performed assessing the *HER2*/CEP17 ratio or the absolute *HER2* copy number, respectively. In the case of the double-signal assay, the recent ASCO/CAP guidelines [19] recommend using the *HER2*/CEP17 ratio to screen for amplified or not amplified breast cancers. However, our data provide another line of evidence that the interpretation of the results of the ISH analyses on interphase nuclei using a dual-signal assay should be performed “with caution”, given the high frequency of complex Chr17 abnormalities involving both CEP17 and *HER2* loci. This has to be taken particularly into account in cases showing an increased number of discrete *HER2* signals, in which the *HER2*/CEP17 ratio may highly impact on the final definition of the gene status, in contrast with cases with *HER2* gene clusters of amplification. The introduction by the ASCO/CAP 2013 of an algorithm that takes into account the ratio first and then the *HER2* gene copy numbers represents an improvement in the identification of HER2 positive tumors by dual-signal assays. We should point out that the sole *HER2* gene copy number method, as used in single-signal assay, best identifies *HER2* gene amplification in interphase nuclei, as CEP17 copy numbers do not reflect Chr17 copy numbers. This finding should be taken particularly into account for those scenarios in which monosomy of Chr17 may be encountered. In this respect the new guidelines seem to be controversial depending on the method of ISH analysis employed. Indeed, by following the single signal copy number method cases with low copy number (<4) are labeled as negative, while the same tumors showing monosomy of Chr17 are labeled as positive if the *HER2*/CEP17 ratio is employed [19–21]. Although on one side the *HER2*/CEP17 ratio may still lead to issues when interpreting Chr17 monosomy and *HER2* copy numbers would be more reliable, on the other hand we should also acknowledge that double signal assays with CEP17 counts may still provide informative parameters. Interestingly, a recent study on patients treated with anthracycline-based chemotherapy in the neoadjuvant setting has shown that CEP17 duplication strongly correlated with higher pCR rates [22] than did *TOP2A* and *HER2*, in both univariable and multivariable analyses. This shows that alteration of CEP17 copy number detectable in interphase nuclei may still represent a prognostic or predictive indicator, although we cannot decipher the real complexity of the rearrangements this chromosome undergoes to. For instance, Chr17 was frequently translocated with chromosome 8 and 11. These chromosomes have been observed in translocations in many breast cancer cell lines [13, 23] and have also been shown to participate in translocation events in cases of primary breast carcinomas [24]. Cytogenetic analysis of primary cultures would be of invaluable help in understanding whether such alterations recapitulate those of primary tumors. In the primary culture here analyzed, chromosome 8 was involved in one of the translocations.

Another finding of our analyses is the invariable co-amplification of *HER2* and *STARD3* on the same metaphase chromosomes. Increased co-amplification of *HER2* and *STARD3* has been described to be correlated with acquired lapatinib resistance [23]. On the other hand, the simultaneous amplification of *HER2* and *TOP2A* was only found in SKBR3 cells, where a pattern of amplification distinct from *HER2* was identified in one of the Chr17 derivatives. This observation provides another line of evidence that, despite the genomic proximity of *HER2* and *TOP2A* and the observation that *TOP2A* amplification seems to be restricted to tumors harboring *HER2* amplification, these two genes are likely to pertain to separate amplicons, as previously suggested [24, 25]. One may speculate that secondary rearrangements may intervene to separate the two genes from the primary amplicons.

## Conclusion

The results of the traditional karyotyping and of FISH and M-FISH assays on metaphase nuclei reported in this study highlight that complex structural alterations of Chr17 encompassing the *HER2* gene and CEP17 are common in breast cancer cell lines. This may reflect the scenario found in breast carcinomas, as this finding was also observed in the primary cell culture raised from a TNBC. Taken together these data indicate that the *HER2*/CEP17 ratio of interphase nuclei, routinely used to select patients for eligibility for anti-HER2 treatment, should be considered with caution and always coupled with the *HER2* gene copy number values in order not to misinterpret *HER2* gene amplification, as recently updated in the ASCO/CAP 2013 [19].

Further investigation on primary cell cultures would be of invaluable help to allow functional analysis in cells harboring Chr17 rearrangements with respect of response to distinct therapies.

## Abbreviations

ATCC: American Type Culture Collection
CEP17: chromosome 17 centromere
CGH: comparative genomic hybridization
Chr17: chromosome 17
ER: oestrogen receptor
PR: progesterone receptor
FISH: fluorescence *in situ* hybridization
IHC: immunohistochemistry
ISH: *in situ* hybridization
LOH: loss of heterozygosity
M-FISH: multi-color fluorescence *in situ* hybridization
SKY: spectral karyotyping
SRA: smallest region of amplification
TNBC: triple negative breast cancer.
